# What matters when examining the performance of salespersons? Analyzing the boundary conditions of personal dispositional factor

**DOI:** 10.3389/fpsyg.2022.1006270

**Published:** 2022-12-05

**Authors:** Muhammad Naeem, Fayaz Ali Shah, Shahid Jan Kakakhel, Shabana Gul

**Affiliations:** ^1^Department of Management Sciences, Islamia College Peshawar, Peshawar, Pakistan; ^2^Virtual University of Pakistan, Lahore, Pakistan; ^3^Institute of Management Sciences, Peshawar, Pakistan

**Keywords:** developmental performance appraisal, evaluative performance appraisal, internal locus of control, external locus of control, job meaningfulness, salespersons, structural equation modeling

## Abstract

**Purpose:**

This study intended to examine the effect of developmental and evaluative purposes of performance appraisal (PA) on job meaningfulness (JM). Furthermore, the study also aimed to assess the moderating effect of personal dispositional factors, that is, internal and external loci of control between developmental and evaluative PA and JM.

**Design/Methodology/Approach:**

A total of 295 questionnaires were received from salespersons working in national and multinational pharmaceutical companies in Khyber Pakhtunkhwa, Pakistan. Data were gathered using a time lag study design using a convenience sampling technique. Data collected *via* questionnaires were analyzed using PLS-SEM to assess measurement and structural models for testing hypotheses.

**Findings:**

Results revealed that developmental PA significantly influenced JM, while evaluative PA failed to influence JM. Furthermore, there was a moderating effect of the external locus of control (ELOC) on the relationship between developmental PA and JM, while the rest of moderating hypotheses failed to influence the relationship of developmental and evaluative PA with JM.

**Practical implications:**

The results can be used as a building block in order to bring positive work outcomes in the form of meaningful work. Organizations should use their PA as a development tool, instead of instrumental or evaluative PA, for making the work more meaningful to the employees.

**Originality/Value:**

The extant literature is limited in terms of assessing the dimensions of PA (developmental and evaluative) in predicting workplace outcomes. Also, examinations of multidimensions of the locus of control are limited in the existing literature between HR practices and work outcomes. The current study has filled these gaps in the contemporary literature.

## Introduction

Human resource plays a key role in today’s competitive environment as it is an important asset for an organization that cannot be copied or replaced. Organizations have realized and recognized the important contribution played by a human being in achieving organizational goals (Babar et al., 2022). Developing HR requires organizations to effectively manage and implement HR practices, that is, recruitment and selection, training and development, performance management, and compensation management (Naeem et al., 2017). Among these practices, performance management plays a key role in evaluating and developing employees for better performance (Naeem et al., 2021). According to Spicer and Ahmad (2006), performance management includes performance appraisal (PA), which is related to evaluating the performance of employees at an individual level. They further argued that PA is used as a tool for assessing employees’ performance and effectiveness against some set standards. PA is operationalized by scholars differently depending upon the nature of their studies and the context in which it is considered (Ho and Kuvaas, 2020). Grubb (2007) argued that different terminologies have been associated with PA, such as performance rating, merit rating, personal rating, performance assessment, and performance evaluation; however, the main purpose behind this HR practice remained the same. Aly and El-Shanawany (2016) commented that for PA to positively influence employees’ attitudes and behaviors, employees must be satisfied with PA conducted in the organization as dissatisfaction with PA will lead to negative attitudes and behaviors such as high turnover intentions (Naeem et al., 2017) and absenteeism (Iqbal et al., 2022).

The extant literature has comprehensively examined the phenomenon of PA and its importance in predicting workplace outcomes; however, numerous queries need to be unearthed. There exists a dearth of literature as to whether PA should be used by organizations as an evaluative PA and how it influences the workplace outcome in the form of job meaningfulness (JM). PA serves three different purposes: developmental, evaluative, and role definition. The first two are related to individuals, while the latter is related to positions or roles of employees in an organization (Youngcourt et al., 2007). However, in this study, the researcher has used the purpose of PA related to individuals, while the position-oriented purpose is beyond the scope of this study. Developmental PA emphasizes skills improvement, career growth, and future development, while evaluative PA mainly focuses on assessing performance against some set standards, or with the employees’ previous year performance (Eyoun et al., 2020).

The literature has predominantly evaluated HR practices in achieving competitive advantage; however, understanding PA along with its dimensions has been rarely explored (Naeem et al., 2021). A previous study pointedly considered a linear relationship between PA and workplace outcomes (Ahmad et al., 2010). Until now, very little is known about the dimensions of PA (Lu et al., 2018; Naeem et al., 2021), and the relationship between PA workplace outcomes is not simple and direct. However, it is subject to certain individual-level factors such personal disposition factors. Among them is the locus of control which is considered predominant in collectivistic and developing countries like Pakistan. Therefore, it is deemed vital to study the boundary conditions of the internal locus of control (ILOC) and external locus of control (ELOC) by which PA influences employees’ attitudes and behaviors (Kuvaas, 2006). The motivation behind the current study is to address these research gaps in the existing literature.

### Context of study

In Pakistan, as per the IMS report, the total worth/size of this industry is $3.10 billion, while the total size is more than $1 trillion in the global market, which is hardly equating to 0.5% of the sector. The sales of pharma products have observed a significant high change over time. From 2012 to 2017, the annual growth rate was reported around 10 to 12%. In addition to these changes, the average increase in the national and multinational firms has been varied; however, taken as a whole, the pharmaceutical industry has shown significant growth (PPMA, 2017).

In addition, related to the total number of workforces working in the industry, there is a difference in the figures; this industry employed around 90,000 employees directly and 150,000 employees indirectly. The overall turnover rate of employees in this industry is low for the top 100 companies. The reason behind the low turnover rate is that these companies offer competitive packages along with other perks and facilities for retaining employees for themselves. Also, pharmaceutical companies offer training activities on a regular basis to polish their employees’ skills and capabilities. This industry in Pakistan is significantly contributing to the tax revenue of the government. This industry is active in terms of philanthropic activities and undertakes initiatives to substantially contribute toward the people’s socioeconomic wellbeing. Altogether, this industry not only pays its taxes to the government; nevertheless, it also contributes at the individual level (PPMA, 2017). Therefore, this sector is important to study in terms of HR practices and how much they predict individual workplace outcomes.

This study aimed to unfold the causal mechanisms of developmental and evaluative PA in explaining JM. Furthermore, this study also intended to examine the boundary conditions of personal dispositional factors such as multidimensional locus of control (internal and external) since the extant literature is limited in terms of examining the importance of different dimensions of PA (Ismail and Gali, 2017). This study is important in a manner that it provides useful insights for understanding the purpose of PA in predicting positive workplace outcomes, such as JM.

This study offers several theoretical contributions to the existing literature: first, to increase our understanding of the dimensions of PA in predicting JM and second, to examine boundary conditions of important personal dispositional factors such as internal and external loci of control in between developmental and evaluating PA and JM. JM has been defined as “the degree to which the employee experiences the job as one which is generally meaningful, valuable, and worthwhile” (Hackman and Oldham, 1976, p. 162).” The internal locus of control (ILOC) has been defined as “individuals’ ability to determine the outcome of their behavior” (Rotter, 1954, p. 466),” while the external locus of control (ELOC) is “an expectancy that personal permanent characteristics and goal-directed behavior are instrumental in obtaining a reinforcement, and the expectancy that outcomes of life events, whether positive or negative, are controlled by powerful others, chance, or luck” (Ibid, p. 33). Furthermore, this study empirically contributes to the existing literature by conducting this appraisal among salespeople of the pharmaceutical industry in Khyber Pakhtunkhwa, Pakistan.

The social exchange theory (SET) has been used as an underpinning theory to explain linkages among constructs considered in this study. SET revealed a mutual relationship between the organization (one party) and employees (second party). When an organization undertakes activities beneficial for employees, it will, in turn, create a feeling of being valued or worthy in employees’ minds (Aryee et al., 2002). Subsequently, when line managers or supervisors utilize PA of employees as a development tool (focus on improving current performance, providing rewards to good performers, and improving the existing performance of employees), it will lead to emerging positive job outcomes in the form of JM. However, when an organization employs PA for the evaluative purpose, the focus of the organization is on evaluating current employees’ performance to reward employees based on their performance. The basic tenet is when an organization deploys PA for the evaluation purpose, employees whose performance match with the desired output or performance are rewarded, while employees who struggle in delivering the desired output or performance are penalized in the form of salary deduction and holding promotion opportunities. Therefore, when organizations exercise PA for evaluative purposes, it will lead to reduced work meaningfulness. Hence, SET is used as an underpinning theory for this study. The next section of the article sheds light on the relevant literature for explaining linkages among constructs considered in this study, followed by an appropriate methodology explaining participants and the procedure of data collection and the analysis technique. The final section of the article highlights the conclusion, discussing findings in light of the previous literature and limitations of the study.

## Literature review

### Performance appraisal dimensions

The purpose of PA has been classified into two types: developmental and evaluative purposes. The developmental purpose of the PA focuses on improving employees’ work skills and experiences, including identifying individual’s strengths and weaknesses and identifying training needs. On the other hand, the evaluative purpose of the PA is concerned with comparing an individual’s performance to a set standard, to the individual’s previous performance, or to the performance of other organizational members” (Lu et al., 2018, p. 3).

Boswell and Boudreau (2002) call developmental PA motivational and formative. When PA is development-oriented, the aim of the organization is on competence, skills growth, and prospect (Youngcourt et al., 2007). Developmental PA gives information based on which an organization determines the training and development needs of employees. Developmental PA identifies individuals’ requirements apart from their development. The extant literature has revealed that using SET as a theoretical lens, focusing on individuals’ development, will create satisfaction and positive behavior at the workplace (Kuvaas and Buch, 2018). Using PA for the developmental purpose of employees, an organization provides performance feedback and identifies career goals progression and paths. As stated earlier, PA provides counseling and coaching to employees to help them achieve their goals. If PA is designed effectively, developmental PA can give a broader framework for developing the skills of employees. It enriches the PA perspective and views, and the information gathered from PA can be used for decision-making in a diverse range of HR disciplines. Developmental PA at the individual-level intends to manage and facilitate employees, while at the organizational level, manages employees in shaping their positive attitudes and behaviors, that is, motivation, commitment, innovative behavior.

Evaluative PA has been commonly used as judgmental, administrative, personnel, or accountability in the literature (Harrison and Goulding, 1997; Poon, 2004). According to Murphy and Cleveland (1995) when an organization intends to use evaluative PA, the purpose is to assess the performance of individuals, groups, or teams and to differentiate their performance from each other. Organizations decide about the salary, promotion, probation, and lay-offs based on their performance.

Schweiger and Sumners (1994) argued that a sound PA system can minimize the chances of lawsuits by resolving employees’ concerns and addressing negative perceptions about PA usage. Havard (2002) commented that if the purpose of PA is communicated to employees being evaluated regarding their expectations and responsibilities, PA will be regarded as a useful activity. Employees need to accept the assessment received from raters after discussing the outcomes of the assessment, both the rater and ratee need to accept and support the PA system for the smooth function of HR practices (Stewart and Stewart, 1977).

The main purpose of PA is to assess and improve employee performance. PA is based on assessing previous performance to identify weak performers and recognize good performers. The PA process is used to set objectives for employees for the coming year based on their potential and relative worth in the organization or department (Schweiger and Sumners, 1994). PA assists in setting goals, and employees are expected to achieve those agreed upon set objectives.

### Job meaningfulness

Employees aim to have meaningful work and careers during their employment in their organization, rather than simply spending time and earning money. People want their career to be meaningful and should mean something (Steger et al., 2012). According to Thakor and Joshi (2005), when organizations care and value employees’ contribution, their job becomes meaningful to them. Hackman and Oldham (1976) defined JM as “the degree to which the employee experiences the job as one which is generally meaningful, valuable, and worthwhile” (p. 162). It also shows the significance of work that attracts employees toward work. Albrecht et al. (2021) defined meaningful work as a “positive, important, useful contribution to a worthwhile purpose through the execution of their work” (p. 238). However, there is no agreed upon definition of meaningful work which is recognized globally. However, in this study, the researcher has used Hackman and Oldham’s (1976) operationalization of the construct.

Thakor and Joshi (2005) commented that when employees consider their jobs meaningful and important, then it will bring positive workplace outcomes in the form of higher motivation, job satisfaction, and work performance. In addition, Jaramillo et al. (2013) found that when marketing staff’s job is more demanding and meaningful, then it will lead to reduction in negative workplace outcomes, such as stress, performance, and turnover intention. Jaramillo et al. (2013) further contended that when an organization follows proper ethical guidelines and sets norms for violating organizational practices, then it will bring clarity in employees’ minds about the employment, thus leading to making the job meaningful for them. When an employee believes that working on an organization is strictly monitored under certain guidelines, it will make the job worthy and the working more meaningful.

### Performance appraisal and job meaningfulness

Employees aim to have meaningful work and careers during their employment in their organization, rather than simply spending time and earning money. People want their career to be meaningful and to mean something (Steger et al., 2012). According to Thakor and Joshi (2005), when organizations care and value employees’ contribution, their job becomes meaningful to them. Hackman and Oldham (1976) defined JM as “the degree to which the employee experiences the job as one which is generally meaningful, valuable, and worthwhile” (p. 162). It also shows the significance of work that attracts employees toward work. Albrecht et al. (2021) defined meaningful work as a “positive, important, useful contribution to a worthwhile purpose through the execution of their work” (p. 238). While there is no agreed upon definition of meaningful work which is recognized globally. However, in this study the researcher has used Hackman and Oldham’s (1976) operationalization of the construct and has been considered as workplace outcomes predicted by developmental and evaluative PA. Relationship between HR practices and workplace outcomes has been reported in the following paragraphs.

Employees’ perceptions about PA have been studied previously in different settings along with its impact on employees’ outcomes as, using a mixed method approach, Bekele et al. (2014) found a significant influence of employees’ PA on work performance, affective commitment, and turnover intention. Similarly, it was also found that PA used for the development perspective has a positive association with behavioral outcomes, such as motivation improvement, work performance, affective commitment, turnover intention (Fakhimi and Raisy, 2013), and employee wellbeing (Ahmad et al., 2022), validating the theoretical assumption of SET, whereby employees feel that their existing organization cares and values there work, which will lead to obligation in the mind of employees to repay their organization by exerting extra effort to improve organizational productivity.

Evidence from the developing country documents that if the employees are satisfied with the existing PA, then such HR practice will significantly predict positive work outcomes in the workplace (Naeem et al., 2017; Ahmad et al., 2022). The study was conducted for explaining the relationship between employees’ perceptions about PA and employee outcomes, such as work effort, affective organizational commitment, and turnover intention. The findings revealed that employees’ perception of PA has a positive influence on the perceived work effort of employees and affective organizational commitment, while PA was negatively influencing turnover intention in the context of higher educational institutes in Pakistan.

Similarly, Kuvaas (2006) conducted a study in a Western context related to PA satisfaction and employee outcomes. Findings posited that PA satisfaction is positively correlated with perceived work performance and affective commitment, while inversely correlated with turnover intentions of employees. Employees satisfied with the current PA will have lesser intentions to quit their existing organization. A similar study was replicated in the Asian context by Vignaswaran (2008) in which he took intrinsic motivation as a mediator in examining the relationship between PA satisfaction and work performance, affective organizational commitment, and turnover intention. His findings depicted that there was a significant impact of PA on workplace outcome variables, and that intrinsic motivation fully mediated work performance and partially mediated affective organizational commitment.

The literature is diverse in terms of the influence of PA in predicting workplace outcomes. However, the extant literature is limited in terms of different purposes of PA and its influence on workplace outcomes. Therefore, we can theorize the following research hypotheses:

*H1: Developmental PA significantly predicts job meaningfulness.*

*H2: Evaluative PA significantly predicts job meaningfulness.*

### Moderating effect of locus of control in between performance appraisal and job meaningfulness

Performance appraisal in predicting positive workplace outcomes has been examined in the extant literature (Kuvaas, 2008; Ismail and Gali, 2017; Naeem et al., 2017). The major purpose of PA according to Eyoun et al. (2020) include the developmental and evaluative or administrative purpose of PA as these purposes are related to individuals working in organizations while the role-definition purpose is associated with the position held by employees which is beyond the scope of this study. They have considered developmental and evaluative PA and studied its influence on psychological contract along with the moderating effect of generational differences (X and Y). Their findings portray that generation Y influences the effect of administrative PA on psychological contract; however, generational differences did not hold the moderating effect between developmental PA in predicting the psychological contract.

Aube et al. (2007) examined the moderating effect of the work locus of control between perceived organizational support and organizational commitment. The work locus of control includes the external LOC, which shows that decisions are influenced by an external environment. The results revealed that the work LOC significantly influences the effect of perceived organizational support and affective commitment. Similarly, the LOC has been investigated between work-related stress and job burnout (Schmitz et al., 2000). The findings reported that employees having the ELOC have lower job satisfaction, while the ELOC positively influences role conflict and role ambiguity.

Kuvaas (2006) explored the moderating effect of intrinsic motivation between PA and work outcomes. It was found that intrinsic motivation moderates the relationship between PA satisfaction and work performance. The moderation analysis was positive for employees having a high level of intrinsic motivation, negative for employees having a lower level of intrinsic motivation.

The aforementioned study revealed that the effect of HR practices, such as PA and workplace outcomes, are subject to certain individual-level factors such as personality traits. It is important to study the individual-level factors between PA and work outcomes. Hence, we can hypothesize the following moderating hypotheses:

*H3(a): Internal LOC moderates the relationship between developmental PA and job meaningfulness such that the relationship will be stronger at a high internal LOC than at a low internal LOC*.

*H3(b): External LOC moderates the relationship between developmental PA and job meaningfulness such that the relationship will be stronger at a low external LOC than at a high external LOC.*

*H4(a): Internal LOC moderates the relationship between evaluative PA and job meaningfulness such that the relationship will be stronger at a high internal LOC than at a low internal LOC.*

*H4(b): External LOC moderates the relationship between evaluative PA and job meaningfulness such that the relationship will be stronger at a low external LOC than at a high external LOC.*

## Research methodology

### Participants

Salespeople working in the pharmaceutical industry have been approached for data collection in Peshawar, Khyber Pakhtunkhwa, Pakistan. Data were collected from sales employees working in local and multinational organizations. This industry has grown substantially over the last two decades. The literature revealed that the total number of pharmaceutical companies has increased significantly (Waheed et al., 2018). In addition, this industry is suitable in terms of understanding and implementing HR practices in their organizations and has contributed well to the economic stability of the country (Waheed, 2017). This study included only sales employees of the industry while ignoring the technical, administrative, and production department employees. The reason behind this is that the appraisal system for evaluating employees’ performance is uniform. Their performance is assessed against the set targets necessary for their career growth, bonuses, promotion, and commissions. Therefore, it was deemed appropriate to approach those respondents who are gauged against uniform criteria of PA.

### Procedure

Data from salespeople were obtained using a structured questionnaire utilizing the time lag study design. Each employee was assigned a separate code to identify them easily in the second round of the data collection phase. Initially, data regarding demographic characteristics, developmental and evaluative PA, ILOC, and ELOC were obtained. After 2 weeks, the respondents were approached again to collect data relating to JM.

The respondents were briefed about the purpose of study, and data were collected in two waves. They were also assured regarding data confidentiality and individual anonymity to make them comfortable in the data collection phase. Initially, 400 respondents were contacted to provide data related to this study. Respondents were enthusiastic in the first round of data collection, and the researcher could obtain 345 questionnaires. The same respondents were again approached using the specific code after 2 weeks. However, the researcher managed to gather 295 questionnaires from the respondents, making a response rate of 73.7%. The researcher was confident enough as the obtained sample size was far above the minimum sample size using power analysis. Table 1 reveals the time lag details of respondents during data collection and the necessary data collected in each phase.

**TABLE 1:** Time lag design.

Constructs	Time lag	Survey reporting
Demographics	T1	Salespersons
Developmental PA	T1	Salespersons
Evaluative PA	T1	Salespersons
Internal LOC	T1	Salespersons
External LOC	T1	Salespersons
Job meaningfulness	T2	Salespersons

### Measures

Questionnaires were administered in English as it is the official language in Pakistan. The questionnaire was divided into three parts: The first part covers the research title, objectives of the study, respondents’ confidentiality, and ensuring anonymity; the second part includes demographic characteristics of respondents, such as age, gender, marital status, qualifications, designation, and work experience; and the third part includes statements related to the main phenomenon of the study under consideration.

All the items for measuring constructs in the survey were collected using a 7-point Likert scale, starting from 1 as “strongly agree” to 7 as “strongly disagree.” The questionnaire contained items about developmental PA, evaluative PA, ILOC, ELOC, and JM.

#### Developmental and evaluative performance appraisal

Boswell and Boudreau’s (2000) tool has been used for measuring the dimensions of PA. Developmental PA was measured through five items as “PA of my organization identifies individual strength.” On the contrary, evaluative PA was measured through four items including a sample item as “PA of my organization guides promotion of an employee.”

#### Internal and external performance appraisal

For measuring internal and external LOC, the famous Spector’s (1988) tool was adopted. Both internal and external LOCs were measured using eight items, respectively. A sample item for the ILOC is “A job is what you make of it” while a sample item for ELOC is “getting this job is mostly a matter of luck.”

#### Job meaningfulness

JM has been measured using 10 items from the work of Steger et al. (2012). A sample item includes “I understand how my work contributes to my life’s meaning.”

### Dealing with common method bias

In quantitative studies, the researchers emphasized how to deal with common method bias (CMB) as it is considered a major limitation in using the survey method (Podsakoff et al., 2003). There are two techniques for dealing with CMB in the extant literature, namely, procedural approach and statistical approach. The researcher followed both techniques to minimize the occurrence of CMB. In the procedural approach, the researcher explicitly promised respondents regarding their confidentiality and anonymity and ensured that data will only be used in an aggregate form. In addition, for the statistical approach, the researcher used the guidelines recommended by Kock (2015) using a full collinearity test. Table 2 reveals the results that variance inflation factor (VIF) values are lower than the threshold value of 3.3, suggesting there is no major issue of CMB. Apart from these approaches, the researcher gathered data from respondents using a time lag research design (using two lags), thus minimizing the occurrence of CMB. Therefore, in this study, CMB is of no major concern.

**TABLE 2:** Inner variance inflation factor (VIF) values.

	JM
DPA	2.944
ELOC	1.182
EPA	2.572
ILOC	2.375

### Control variables

The ANOVA test was used to assess the impact of demographic variables on the endogenous construct of the study. Control variables including age, gender, marital status, qualification, and experience are not part of the research study, although they substantially impact the findings, thus leading to a false conclusion (Naeem et al., 2021). The findings of the ANOVA test revealed an insignificant impact of demographic variables on JM (endogenous construct). Hence, demographic characteristics were ignored from further analysis.

## Results

Partial least square structural equation modeling (PLS-SEM) was used as the analytical approach for analyzing the findings of this study. SmartPLS version 3.2.9 was used for testing measurement and structural models. The extant literature significantly relies on using PLS-SEM for data analysis (Memon et al., 2019; Naeem et al., 2021). There are several reasons behind using a variance-based approach. The data characteristics such as smaller sample size, providing highly robust analysis even missing values exist in data, and being non-parametric technique, PLS-SEM does not take into account the normality assumption. Concerning model characteristics, PLS-SEM can easily handle complex models having complex paths (Hair et al., 2017). There are two models for running PLS-SEM. First is the measurement model used for reporting the validity and reliability of the questionnaire. The measurement model includes the value of factor loadings, composite reliability (CR), Cronbach’s alpha, and average variance extracted (AVE). The second is a structural model used for hypothesis testing by providing the values of path coefficients, standard deviation, t-statistics, and *p*-values essential for accepting or rejecting hypotheses.

### Preliminary analysis

Before addressing validity, reliability, and hypothesis testing, the researcher ran exploratory factor analysis (EFA) to assess the unidimensionality of constructs and sampling adequacy and consistency. The Kaiser–Meyer–Olkin (KMO) test gives a value of 0.80, which is above 0.50, representing the adequacy of the sample. Furthermore, Bartlett’s test of sphericity (BTS) values were significant (*p* < 0.01), affirming data suitability. However, there were three items identified during EFA, which failed to load well onto their respective constructs and were deleted: DPA2, ILOC4, and JM3.

### Descriptive statistics

Table 3 reveals the personal characteristics of respondents who were approached during the data collection phase. A majority of respondents were male (*n* = 276), with a substantial portion of them being married (*n* = 168). Majority of respondents were middle-aged (*n* = 183), with a maximum age bracket of 20–29 years (*n* = 190). Most of the respondents were well-educated and had bachelor’s degrees (*n* = 133), and a reasonable work experience of 2–5 years (*n* = 182). Table 4 represents the average value, standard deviation, and association among the constructs considered in this study.

**TABLE 3:** Respondents’ details.

Respondent details	Count	(%)
**Gender**		
Male	276	93.6
Female	19	6.4
**Marital status**		
Unmarried	127	43.1
Married	168	56.9
**Designation**		
Top level	19	6.4
Middle level	183	62
Lower level	93	31.5
**Age of respondents**		
20–29	190	64.4
30–39	84	28.5
40–49	16	5.4
50–59	5	1.7
**Qualification**		
Matric	14	4.7
Intermediate	15	5.1
Bachelor	133	45.1
Master	112	38
M.Phil./MS	21	7.1
**Work experience**		
2–5 years	182	61.7
6–10 years	59	20
11–15 years	29	9.8
Above 15 years	25	8.5

*N* = 295.

**TABLE 4:** Mean, standard deviation, and correlation matrix.

Constructs	Mean	SD	1	2	3	4	5	6	7	8	9	10	11
1. Gender	–	–	1										
2. Marital status	–	–	−0.079	1									
3. Designation	–	–	−0.092	−0.245**	1								
4. Age of respondents	–	–	−0.029	0.348**	−0.373**	1							
5. Qualification	–	–	0.045	0.194**	−0.282**	0.119*	1						
6. Work experience	–	–	−0.120*	0.281**	−0.282**	0.622**	0.124*	1					
7. DPA	5.649	1.138	−0.135*	0.076	−0.074	0.055	0.057	−0.019	**(0.894)**				
8. EPA	5.215	1.211	−0.108	0.077	−0.001	0.064	0.087	0.040	0.582**	**(0.802)**			
9. ILOC	5.541	0.985	−0.088	−0.006	−0.103	0.008	−0.055	−0.077	0.638**	0.495**	**(0.851)**		
10. ELOC	4.363	1.548	0.100	−0.065	0.197**	−0.126*	−0.046	0.028	0.151**	0.389**	0.289**	**(0.934)**	
11. JM	5.475	1.197	−0.114	0.091	−0.086	0.143*	−0.003	0.106	0.642**	0.497**	0.710**	0.245**	**(0.931)**

*Correlation is significant at the 0.05 level (2-tailed). **Correlation is significant at the 0.01 level (2-tailed). Bold indicates the values of internal consistency reliability.

### Measurement model

The measurement model represents construct validity, reliability, convergent, and discriminant validity (DV). For construct reliability, Memon et al. (2019) recommended reporting internal consistency reliability (ICR), instead of Cronbach’s alpha value. ICR represents “the degree to which the items measure latent construct” (Memon et al., 2019; p. 1058). Convergent validity (CV) as per Hair et al. (2017) is defined as “the extent to which a measure correlates positively with alternative measures of the same construct” (p. 112). CV has been presented *via* outer loadings and average variance extracted (AVE). An AVE value of 0.50 or above is desirable for the construct to establish CV. The findings (Table 5) revealed reasonable AVE values, DPA (0.679), EPA (0.513), ILOC (0.534), ELOC (0.639), and JM (0.627). Hence, CV is established in this study.

**TABLE 5:** Items loading, composite reliability (CR), and convergent validity (CV).

Constructs	Items	Loadings	rho_A	CR	AVE
Developmental PA	DPA1	0.797	0.850	0.894	0.679
	DPA3	0.806			
	DPA4	0.872			
	DPA5	0.817			
Evaluative PA	EPA1	0.873	0.765	0.802	0.513
	EPA2	0.563			
	EPA3	0.809			
	EPA4	0.564			
Internal LOC	ILOC2	0.769	0.790	0.851	0.534
	ILOC3	0.729			
	ILOC6	0.750			
	ILOC7	0.682			
	ILOC8	0.719			
External LOC	ELOC1	0.768	0.985	0.934	0.639
	ELOC2	0.801			
	ELOC3	0.758			
	ELOC4	0.788			
	ELOC5	0.760			
	ELOC6	0.822			
	ELOC7	0.789			
	ELOC8	0.899			
Job meaningfulness	JM1	0.819	0.916	0.931	0.627
	JM2	0.788			
	JM4	0.752			
	JM6	0.809			
	JM7	0.751			
	JM8	0.846			
	JM9	0.774			
	JM10	0.791			

Items deleted due to low loadings: ILOC1, ILOC5, and JM3.

Furthermore, Hair et al. (2017) defined DV as the “extent to which a construct is truly distinct from other constructs” (p. 115). In other words, DV shows that the items measuring one construct are substantially different from the items measuring another construct considered in a study. Henseler et al. (2015) recommended the use of heterotrait-to-monotrait (HTMT) ratio as it is a more robust and up-to-date technique for assessing DV. However, HTMT values revealed that EPA lacks DV as its value is greater than the threshold value (0.90). Thus, the researcher opted for other techniques to check whether EPA has established DV, that is, cross-loadings. Table 6 reveals the cross-loadings value for each item, confirming that all constructs considered in this study have achieved DV. Therefore, using cross-loading, the researcher can cautiously claim that DV was established in this study.

**TABLE 6:** Cross-loadings.

Items	DPA	ELOC	EPA	ILOC	JM
DPA1	**0.797**	0.203	0.596	0.542	0.559
DPA3	**0.806**	0.021	0.566	0.524	0.437
DPA4	**0.872**	0.178	0.709	0.554	0.583
DPA5	**0.817**	0.202	0.598	0.628	0.600
ELOC1	0.177	**0.768**	0.288	0.209	0.194
ELOC2	0.198	**0.801**	0.266	0.283	0.276
ELOC3	0.032	**0.758**	0.093	0.070	0.100
ELOC4	0.087	**0.788**	0.210	0.177	0.120
ELOC5	0.126	**0.760**	0.218	0.253	0.165
ELOC6	0.155	**0.822**	0.297	0.275	0.270
ELOC7	0.032	**0.789**	0.278	0.099	0.092
ELOC8	0.222	**0.899**	0.328	0.317	0.364
EPA1	0.723	0.188	**0.873**	0.603	0.581
EPA2	0.317	0.301	**0.563**	0.270	0.264
EPA3	0.650	0.204	**0.809**	0.505	0.527
EPA4	0.320	0.387	**0.564**	0.327	0.300
ILOC2	0.478	0.289	0.429	**0.769**	0.610
ILOC3	0.457	0.212	0.410	**0.729**	0.607
ILOC6	0.556	0.182	0.505	**0.750**	0.566
ILOC7	0.634	0.113	0.573	**0.682**	0.479
ILOC8	0.365	0.318	0.381	**0.719**	0.372
JM1	0.489	0.286	0.468	0.599	**0.819**
JM2	0.527	0.203	0.444	0.594	**0.788**
JM4	0.465	0.218	0.457	0.568	**0.752**
JM6	0.535	0.244	0.518	0.526	**0.809**
JM7	0.553	0.164	0.515	0.583	**0.751**
JM8	0.574	0.276	0.559	0.649	**0.846**
JM9	0.577	0.239	0.498	0.617	**0.774**
JM10	0.509	0.234	0.455	0.539	**0.791**

Bold indicates factor loadings.

### Structural model

It assesses the linkages among constructs considered and analyzes the predictability of hypothesized models (Hair et al., 2017). To calculate beta values, *t*-values, and *p*-values, the researcher used the 5,000 resamples bootstrapping method to obtain the results. R-square reveals the predictability power of a model, and its recommended value is equal or above 0.1. The findings revealed an R-square of 0.662, which signifies the model predictive capacity.

Table 7 shows the result of the proposed relationship among the constructs considered in this study. The findings revealed that DPA significantly influences JM (β = 0.171, *t* = 2.242, *p* < 0.013), and the confidence interval lower limit and upper limit do not contain zero in between. Thus, H1 is empirically supported. However, EPA failed to have a significant influence on JM (β = 0.034, *t* = 0.425, *p* > 0.05), while the confidence interval lower limit and upper limit do contain zero in between, thus rejecting H2 statistically.

**TABLE 7:** Results of structural model.

Path	β	SD	*t*-value	*P-value*	CI lower limit	CI upper limit	Decision
DPA - > JM	0.171	0.076	2.242	0.013	0.047	0.293	Supported
EPA - > JM	0.034	0.080	0.425	0.335	−0.090	0.174	Not supported
DPA*ILOC - > JM	0.030	0.083	0.360	0.359	−0.100	0.172	Not supported
DPA*ELOC - > JM	−0.221	0.098	2.262	0.012	−0.378	−0.064	Supported
EPA*ILOC - > JM	0.040	0.095	0.419	0.338	−0.120	0.192	Not supported
EPA*ELOC - > JM	−0.098	0.096	1.015	0.155	−0.228	0.089	Not supported

Moderating hypotheses were tested using the product indicator approach as all the constructs considered in this study were reflectively measured. Moderating hypotheses were postulated to examine the influence of internal LOC and external LOC between DPA and JM. The findings portray that the internal LOC failed to moderate the relationship between DPA and JM (β = 0.030, *t* = 0.360, *p* > 0.05), thus rejecting H3(a) statistically. However, ELOC moderates the relationship between DPA and JM (β = −0.221, *t* = 2.262, *p* < 0.05), thus statistically supporting H3(b).

**FIGURE 1:**
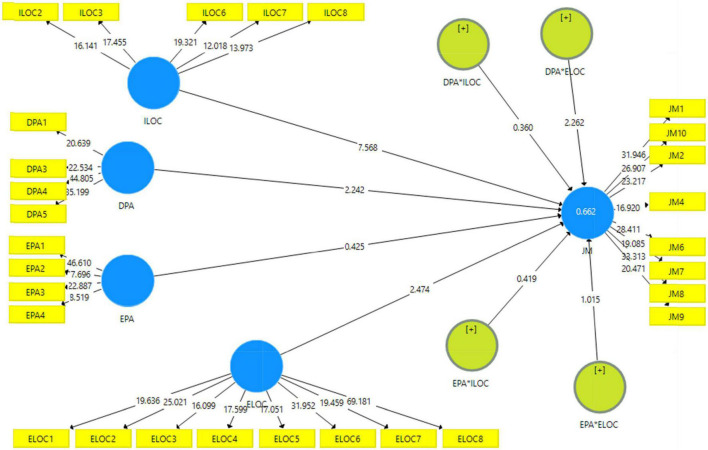
Structural model assessment.

Furthermore, the moderating effect of the ILOC and ELOC between EPA and JM was assessed. Table 7 reveals that the ILOC failed to moderate the relationship between EPA and JM (β = 0.040, *t* = 0.419, *p* > 0.05), thus rejecting H4(a) statistically. Similarly, the ELOC also failed to influence the relationship between EPA and JM (β = −0.098, *t* = 1.015, *p* > 0.05). Hence, H4(b) was statistically rejected. The structural model assessment has been portrayed in Figure 1.

### Effect size *(f^2^)* and predictive relevance (Q^2^)

In this study, an endogenous construct was predicted by more than one exogenous construct (DPA and EPA). Therefore, the researcher deemed it necessary to estimate the effect sizes of individual exogenous constructs. Ali et al. (2016) defined the endogenous construct as “the extent to which a predicting (exogenous) variable contributes to the R^2^ value of an endogenous latent variable” (p. 84). As per Cohen’s (1988) criteria, a value of 0.02 represents small effect size, and 0.15 and 0.35 represent medium and large effect size, respectively. Table 8 shows *f*^2^ values of DPA (0.184) and EPA (0.059), representing medium effects of DPA and EPA, respectively.

**TABLE 8:** Effect size and predictive relevance.

	*f* ^2^	*Q* ^2^
DPA - > JM	0.184	
EPA - > JM	0.059	
JM		0.289

Furthermore, R^2^ shows the within-sample predictive capacity of a research model, while Chin (2010) suggested using the predictive sample reuse technique (Q^2^) for analyzing the predictive relevance of a model. Using the blindfolding technique, Q^2^ evaluates the model predictive validity by removing data for certain items and using the remaining data for predicting the omitted parameters. A Q^2^ value above zero denotes a predictive relevance of a model, and vice versa (Richter et al., 2016). Table 8 shows a Q^2^ value above zero, indicating that the model has predictive validity.

## Discussion

This study has investigated the effect of developmental PA and evaluative PA on JM. Also, the study aimed to assess the moderating effect of personal dispositional factors (internal LOC and external LOC) between developmental and evaluative PA in explaining JM. The findings of study revealed that developmental PA significantly influenced JM, while evaluative PA failed to influence JM in the pharmaceutical industry in Khyber Pakhtunkhwa, Pakistan. Furthermore, moderation analysis holds in the case of the external LOC. The external LOC only moderates the influence of developmental PA on JM, while the rest of the hypotheses was rejected statistically, and the phenomenon does not prevail in developing country’s context like Pakistan. The possible reasons for the rejection of hypothesis could be the collectivistic culture of Pakistan, wherein people share their concerns with others. Personal factors do not significantly influence the relationship between HR practices and work attitudes or behaviors.

When employees perceive that an organization intends to use PA for career advancement, future development of employees, and skills improvement, then it leads to positive workplace outcomes in the form of job embeddedness, as reported in this study that developmental PA will lead to meaningful work for salespersons in the context of the pharmaceutical industry. On the other hand, the organizational PA system intends to measure the performance of employees based on some set standards, or with their previous performance or assessing their performance against another employees’ performance to evaluate employees’ performance, it will create a sort of envy among salespersons. Therefore, employees will be unable to apprehend their work meaningfully. This study contributed to the existing literature by investigating the effect of developmental and evaluative PA on individual outcomes in the form of JM. Researchers have stressed examining HR practices and employees’ attitudes and behaviors. However, the literature was limited in terms of studying the dimensionality of PA specifically related to individuals, instead of their jobs such as developmental and evaluative PA in influencing workplace attitudes including JM. Furthermore, this study contributed to examining the interaction effect of personal dispositional factors such as internal and external LOC. Scholars such as Eyoun et al. (2020) and Ho and Kuvaas (2020) suggested using personality traits between HR practices and workplace outcomes. Consequently, this study was an attempt to study whether developmental and evaluative PA can significantly explain JM in the context of the pharmaceutical industry. In addition, this study also examined the moderating effect of the internal LOC and external LOC to get insights into whether it influences the relationship between these dimensions of PA and JM.

This study further contributed to the literature by considering the dimensions of PA such as developmental and evaluative PA in predicting JM. Developmental PA influences JM, while evaluative PA has an insignificant influence on JM. The findings of this study contradict with the previous recent literature as Naeem et al. (2021) found a positive and significant impact of developmental and evaluative PA on job embeddedness. Furthermore, this study has considered the locus of control as a multidimensional construct as a moderating construct. Spector (1988) argued that the LOC is rooted in organizational psychology and investigated its moderating effect recommended in the existing literature. This study provides useful insights into examining the LOC in an Asian context, that is, Pakistan. Therefore, this study also empirically substantiated the literature by considering pharmaceutical industry salespeople as there is a dearth of literature investigating such phenomena among sales and marketing employees.

### Managerial implications and theoretical implications

Managers and practitioners can effectively obtain useful insights while implementing PA in their organizations. Managers must make their appraisal system development-oriented, which not only focuses on the career advancement of employees but also bridges the gap between current performance and desired performance, as evident from the findings of this study. Furthermore, when PA is used by organizations as an instrumental tool for the evaluative purpose wherein performance is measured against some set standards for measuring employees’ performance by comparing it with others’ performances and then linking their performance with reward mechanism will likely lead to negative workplace attitudes and behaviors. Employees would think that they are being disgraced in their organization by comparing their performance against their colleagues’ performances. Therefore, managers need to consider negative consequences associated with instrumental usage of PA. Moreover, the appraisal system of an organization should be linked to an individual personality trait. Managers must assess the personality characteristics of an individual employee and then define the PA system according to their employees’ needs.

This study provides several theoretical implications for researchers in the field of organizational behavior and workplace psychology. This study contributes to the existing literature by studying important HR practice, that is, PA along with its two dimensions, namely, developmental and evaluative PA. These two dimensions are important and play a key role in enhancing individuals’ performance since it focuses on individuals, instead of their position or role they are in. Another substantial contribution of this study is to understand the linkage of HR practice with JM. This study has used relatively new constructs, such as JM, which has been ignored in the existing literature as an outcome of HR practices. Previous scholars have extensively used job satisfaction (Poon, 2004), proactive behavior (Jia et al., 2020), affective organizational commitment, turnover intention (Naeem et al., 2017), and engagement (Memon et al., 2020), while the literature is limited in terms of new constructs as the outcomes of HR practices. Furthermore, one of the most important theoretical contributions of this study is to investigate the boundary conditions of the ILOC and ELOC between PA purpose and JM. Hence, studying the multidimensional nature of the locus of control significantly contributes to the existing body of knowledge.

Moreover, this study validated the use of the SET in the context of the pharmaceutical industry of Pakistan as previous scholars have ignored studying this important sector in the Asian context and as the extant literature has predominantly emphasized on the Western context. However, the organizational culture and working context of developing countries are significantly different from those in Western countries. Therefore, it was deemed necessary to study this phenomenon in the given context. Memon et al. (2020) concluded that PLS-SEM is considered an advanced statistical analysis technique for testing complicated models having multiple mediators, moderators, or multidimensional constructs. Previous studies mostly relied on first-generation (Poon, 2004; Kuvaas, 2006; Naeem et al., 2017) statistical tools, while this study has used PLS-SEM for hypothesis testing as it is considered the most robust and advanced approach of SEM in the field of social sciences.

### Future research directions

This study was limited to salespeople working in the pharmaceutical industry in Khyber Pakhtunkhwa. It is therefore important that researchers must be cautious about generalizability of the study. This study has only considered those dimensions of PA that are individual-oriented. Future researchers should consider dimensions related to the position held by employees in their organization. Furthermore, future researchers should expand the boundary conditions from individual-level to organizational-level or group-level to obtain useful insights for future studies. In this study, a sample was drawn from salespeople using a convenience sampling method, which limits generalizability. Therefore, future research should consider probability sampling techniques for obtaining data from respondents. In addition, future studies should consider testing the effects of cultural context on relationship between HR practices and workplace outcomes to find out whether the relationship holds or not.

### Conclusion

This study empirically examined the interaction effect of internal and external loci of control on the relationship between developmental and evaluative PA in predicting JM among the salespersons working in the pharmaceutical industry in Peshawar, Khyber Pakhtunkhwa. Generally, the findings of this study revealed that developmental PA significantly influences JM of employees. The moderating effect was substantiated in the case of the ELOC in between developmental PA and JM, while the ILOC did not moderate the relationship between developmental PA and JM. Furthermore, the moderating effect of internal and external loci of control was insignificant between evaluative PA and JM. Managers need to frame their PA system as a developmental tool as evident from the findings of this study by considering their employees’ self-growth and career advancement. Moreover, linking PA with career growth and providing regular feedback to their employees will lead to more meaningful work.

## Data availability statement

The raw data supporting the conclusions of this article will be made available by the authors, without undue reservation.

## Ethics statement

The studies involving human participants were reviewed and approved by Islamia College Peshawar Ethics Committee. The patients/participants provided their written informed consent to participate in this study.

## Author contributions

MN wrote the preliminary study including data collection and analysis. FS supervised overall the study. SK did the conceptualization of the study. SG assisted in writing literature and methodology section of the manuscript. All authors contributed to the article and approved the submitted version.
